# Research on middle school teachers’ technostress empowered by artificial intelligence

**DOI:** 10.3389/frai.2025.1732088

**Published:** 2026-01-15

**Authors:** Chenguang Wu, Wenlan Zhang, Liangliang Hu, Ming Li

**Affiliations:** 1 Faculty of Education, Shaanxi Normal University, Xi’an, China; 2 Xi’an Chang’an No.1 High School, Xi’an, China

**Keywords:** artificial intelligence, influencing factors, mechanism, middle school teachers, technostress

## Abstract

**Introduction:**

Technostress is an essential factor in predicting middle school teachers’ willingness to adopt artificial intelligence (AI) in future educational practices and their actual use of such technologies. This study examines technostress among middle school teachers in the context of AI integration and explores how personal competence (including digital awareness, digital technology knowledge and skills, and digital application competence), role conflict, organizational support, and technological features influence technostress.

**Methods:**

The Technology Acceptance Model (TAM) is employed as the theoretical underpinning for the present research, using survey data from 301 middle school teachers, a path model was constructed to analyze these relationships.

**Results:**

The results indicate that the overall level of technostress is relatively low; however, different teacher groups experience distinct sources of stress. Specifically, appropriate technological features and strong digital awareness effectively alleviate technostress, while role conflict intensifies it. Furthermore, these factors play a significant mediating role between organizational support and technostress.

**Discussion:**

Based on these findings, the study proposes several strategies to mitigate technostress among middle school teachers. First, a tiered and category based approach should be adopted to provide targeted support according to teachers’ actual needs. Second, it is important to balance the relationship between technological supply and educational demand to ensure sustainable implementation. Third, showcasing typical successful cases can help enhance teachers’ digital awareness and confidence in using AI. Finally, strengthen role positioning and work flexibility to ease teachers’ role conflict. These strategies offer practical guidance for educational administrators seeking to promote the effective integration of AI technologies in middle school education.

## 1 Introduction

Driven by international policies promoting digital transformation in education, AI technologies have gradually permeated all aspects and processes of teaching and learning. This integration is rapidly reshaping educational systems and transforming teaching practices. While AI has infused new vitality into education, it has also introduced considerable technostress among middle school teachers (Yi and Xue, 2022). Such stress often results in occupational burnout and reduced job satisfaction, becoming a significant barrier to improving teaching quality and advancing educational digitalization.

Previous research by Ghanizadeh et al. (2025) has shown that technostress exerts a substantial negative influence on teachers’ professional development. Other studies have revealed that middle school teachers currently experience relatively high levels of stress associated with AI technology use, which serves as a major factor driving their resistance to adopting AI in teaching (Zhang, 2025). However, most existing research on AI implementation in secondary education has focused primarily on technology integration within instructional practices. Few studies have specifically examined technostress among middle school teachers, and even fewer have analyzed the interrelationships among its influencing factors.

Given this gap, there is an urgent need for comprehensive strategies to alleviate teachers’ technostress and facilitate the effective adoption of AI in education. Therefore, this study aims to identify the key factors influencing teachers’ technostress, explore the interaction mechanisms among these factors under the context of AI empowerment, and propose targeted strategies to mitigate technostress among middle school teachers.

## 2 Literature review

### 2.1 Technostress (TS) and teachers’ technostress

Research on technostress has traditionally concentrated on the industrial and governmental sectors, with comparatively little attention paid to the field of education (Li and Wang, 2021), most studies addressing teachers’ technostress were published after 2015, with a particular concentration between 2020 and 2025. Since 2015, emerging technologies such as deep learning and big data have increasingly penetrated educational contexts, reshaping teaching methods and learning environments. The COVID-19 pandemic further accelerated the integration of technology into education, intensifying teachers’ exposure to technological demands (Yang et al., 2025), it exerts a significant adverse effect on individual productivity; in the absence of effective mitigation strategies, it not only negates the productivity-enhancing potential inherently associated with technological adoption but also diminishes individuals’ satisfaction levels with both information and communication technologies utilization and their professional roles (Tarafdar et al., 2007; Ragu-Nathan et al., 2008; Tarafdar et al., 2010; Pullins et al., 2020) a deep and urgent research into technostress among teachers is needed.

Khlaif et al. (2023) defined teachers’ technostress as the physical and psychological strain experienced when adapting to new technologies—such as digital devices, platforms, shifts in teaching models, or other media—during the teaching and learning process. Coklar et al. (2017) conceptualized technostress through five dimensions: learning–teaching process–oriented, profession-oriented, technical issue–oriented, personal-oriented, and social-oriented, and subsequently developed a corresponding technostress scale. Similarly, Ortega-Jiménez et al. (2023). identified four dimensions of teachers’ technostress: skepticism, fatigue, anxiety, and ineffectiveness.

Overall, technostress represents a dynamic phenomenon encompassing both positive and negative outcomes (Tu et al., 2025). However, this study specifically focuses on the adverse effects of technostress among teachers. In this context, technostress is defined as a state of maladaptation experienced by teachers resulting from the ongoing demands of integrating and applying AI technologies in daily educational practice. Grounded in this definition, this study precisely focuses on the middle school teachers, addressing the gap in existing research which often concentrates on higher education or offers generalized discussions on basic education teachers. Furthermore, it delves into the interactive mechanisms among three types of factors: technological features, personal competence, and organizational support. By closely integrating the contextual characteristics of middle school teaching, the study proposes targeted *alleviate* strategies, thereby providing practical support for the deep and beneficial integration of artificial intelligence technology and secondary education.

### 2.2 Technostress among middle school teachers and its influencing factors

In the field of basic education, research has primarily focused on teachers’ instructional performance or pedagogical behavior, often treating technostress as a mediating variable. For example, Feng et al. (2023) examined the effects of technostress on teachers’ job performance and innovative thinking; Li et al. (2023) explored how technostress influences pre-service mathematics teachers’ mathematical cognition; and Güner et al. (2025) investigated the mediating role of technostress between pre-service teachers’ professional identity and their Technological Pedagogical and Content Knowledge (TPACK) competencies. Similarly, Ghanizadeh et al. (2025) adopted a quantitative approach to analyze the impact of technostress on teachers’ work engagement.

From a theoretical perspective, scholars have sought to identify the underlying sources of teachers’ technostress. Zhao et al. (2024) analyzed technostress in the digital-intelligent era from the perspectives of technological essence and the human–technology relationship, while Wang and Li (2022) examined the formation of technostress across cognitive, affective, and behavioral dimensions. Empirical studies focusing on primary and secondary school teachers have also expanded this understanding. For instance, Lee et al. (2025) explored the technostress experienced by middle school physical education teachers when integrating technology and assessed the mitigating role of TPACK competence. Wang et al. (2025) found that teachers’ psychological resilience and administrative support could alleviate technostress. Similarly, Tu et al. (2025) demonstrated that organizational support and improved digital literacy significantly reduce technostress among STEM teachers. Zhao et al. (2022) identified three key sources of technostress—self-development expectations, technology perception, and technical support for technology adoption. Rao et al. (2025) highlighted the effects of workload, role burden, and technical burden on teachers’ digital stress; and Zheng et al. (2025) revealed that school-based support and teachers’ value orientations significantly influence technostress in online learning contexts, drawing on the Person–Environment Fit Theory.

A synthesis of the existing literature indicates that teachers’ technostress primarily arises from three interrelated dimensions: technological factors, personal attributes, and organizational support mechanisms (Yang et al., 2025). Technological factors—such as perceived usefulness, ease of use, exert a substantial influence on technostress levels (Wei et al., 2025; Fei et al., 2025; Christian et al., 2020; Revilla Muñoz et al., 2017). Likewise, personal attributes, including teachers’ attitudes toward technology, self-efficacy, and TPACK competence, play a critical role in shaping their experiences of technostress, whereas at the individual level, proactive coping strategies, including the active reframing of stressful contexts and the enhancement of control over information and communication technologies, are advocated (Pirkkalainen et al., 2019), with the enhancement of technological self-efficacy further identified as a pivotal mediating mechanism for alleviating technostress (Pirkkalainen et al., 2019; Tarafdar et al., 2015; Zito et al., 2021; Xin and Li, 2025; Trua et al., 2023; Li and Wang, 2021). Organizational support, which encompasses institutional policies such as encouraging employee participation, providing training support, promoting technology integration, and offering operational assistance during implementation, also exerts a significant influence on teachers’ technostress levels (Tarafdar et al., 2015; Zito et al., 2021; Revilla Muñoz et al., 2017; Li et al., 2024).

Despite growing research attention, the interactions among these influencing factors have received limited examination. Existing studies remain insufficient in clarifying how specific variables jointly affect teachers’ technostress and the mechanisms through which these interactions occur, the formation mechanism of teachers’ technostress remains insufficiently clear and requires sustained attention (Tu et al., 2025; Yang et al., 2025). Therefore, this study attempts to investigate technostress among middle school teachers, with the following specific objectives: (1) identify the influencing factors of middle school teachers’ technostress; (2) explore the mechanism of action among these influencing factors; (3) provide effective strategies for alleviating middle school teachers’ technostress.

### 2.3 Theoretical framework

To better understand the technostress situation of middle school teachers in the context of AI empowerment, this study adopts the TAM (Davis, 1989) as its theoretical framework. The TAM takes perceived usefulness and perceived ease of use as key variables, which can directly predict usage intention and actual usage behavior. The TAM exhibits robust structural stability, offers straightforward integration of additional variables for high extensibility, and has undergone extensive validation across a wide array of technology adoption scenarios, it provides a compelling explanation for users’ technology adoption behaviors, numerous recent studies on the integration of AI in education have adopted TAM as their theoretical foundation. For example, Runge et al. (2025). explored pre-service teachers’ acceptance of AI based on the TAM. Mulyani et al. (2025) also took TAM as the theoretical basis to study the impact of AI technology on teachers’ teaching performance; this study also takes TAM as its core conceptual framework.

### 2.4 The present study

Unlike previous forms of technology integration, the human–machine interaction capabilities of AI technologies place new demands on teachers’ professional competence and challenge their traditional roles. Consequently, this study investigates the effects of organizational support, teachers’ personal competence (including digital awareness, digital knowledge and skills, and digital application capabilities), role conflict, and technological features on the current technostress experienced by middle school teachers. It also explores the mechanisms through which these factors interact to shape teachers’ technostress.

Accordingly, this study seeks to address the following research questions:

RQ1: What is the current status of technostress among middle school teachers in the context of AI integration?

RQ2: How does technostress differ across various groups of middle school teachers?

RQ3: What factors influence technostress among middle school teachers, and how do these factors interact with one another?

### 2.5 Hypothesis formulation and development

#### 2.5.1 Personal competence of teachers (PC)

For educators, enhancing digital literacy is a pivotal strategy for bridging the digital divide and mitigating the associated digital burdens (Wang, 2025). To better evaluate teachers’ competencies in this context, UNESCO released the ICT Competency Framework for Teachers; The United States released the ISTE Standards for Educators, China’s Ministry of Education has issued the Teachers’ Digital Literacy Framework, which outlines the digital awareness, technological knowledge and skills, and application competencies required for teachers integrating AI into education. Accordingly, this study adopts the Teachers’ Digital Literacy Framework as the foundation for defining teachers’ individual competencies (Ministry of Education of the People’s Republic of China, 2022).

*“Digital awareness (DA)”* refers to teachers’ internalized and dynamic cognitive responses to digital activities. It encompasses three dimensions: digital cognition, digital willingness, and digital will. Digital cognition is consistent with the understanding of information and technology in educational policies as defined by UNESCO, involves understanding the value of digital technologies in educational development and recognizing the opportunities and challenges they bring to education. Digital willingness reflects teachers’ proactive attitude toward learning and utilizing digital resources, consistent with the concept of digital willingness advocated by UNESCO. Digital will denotes the confidence and determination to overcome difficulties and challenges encountered during the digitalization of education (Wu et al., 2023), this concept has not been clearly defined in relevant international educational standards and can be interpreted as a new competency requirement for teachers in the context of China’s educational digital transformation (Pan and Ban, 2023).

This study proposes Hypothesis

*H1.1*: DA can effectively alleviate teachers’ TS.

*Digital technology knowledge and skills (DTKS) encompasses digital technology knowledge and digital technology skills. “Digital technology knowledge”* refers to teachers’ understanding of the fundamental concepts and basic principles of digital technologies, while *“digital technology skills”* encompass the ability to select appropriate digital resources and apply them effectively in educational contexts (Wu et al., 2023). UNESCO defines digital technology knowledge and skills as ‘the application of digital skills’, emphasizing that basic information and communication technology (ICT) skills are a prerequisite for integrating technology into teachers’ professional responsibilities. When teachers possess a solid foundation in digital technology skills and are capable of independently resolving simple technical problems, they can effectively reduce their technostress and enhance their perception.

Therefore, this study makes the hypothesis

*H1.2*: DTKS can effectively alleviate teachers’ TS.

*“Digital application competence (DAC)”* refers to teachers’ ability to conduct educational and instructional activities through the effective application of digital technology resources. This competence includes digital lesson design, digital teaching implementation, and digital assessment practices (Wu et al., 2023). Such competency requirements are highly consistent with the provisions of the UNESCO framework, which mentions “digital application” in multiple modules (e.g., “application of digital skills” and “organization and management”), When middle school teachers lack the ability to use digital technologies to solve instructional problems, their technostress tends to increase. Conversely, enhancing teachers’ digital application competence can effectively alleviate technostress.

Therefore, this study proposes the following hypothesis

*H1.3*: DAC can effectively alleviate teachers’ TS.

#### 2.5.2 Role conflict (RC)

The rise of AI technologies has intensified role conflict among teachers. They are required to navigate not only traditional human–student relationships but also increasingly complex interactions involving human–machine dynamics. Balancing the triadic relationship among teachers, students, and intelligent technologies has expanded teachers’ responsibilities and disrupted their established sense of authority in the classroom. As a result, many teachers experience uncertainty and confusion in redefining their professional roles (Guo and Wu, 2022), high job demands and role ambiguity not only significantly exacerbate teachers’ technostress but also diminish their perception of technological features. (Salanova et al., 2013; Labarthe-Carrara et al., 2024).

Therefore, this study proposes Hypothesis

*H2.1*: Teachers’ RC will increases teachers’ TS.

*H2.2:* Teachers’ Role Conflict RC will diminish their perception of Technical Features TF.

#### 2.5.3 Technological features (TF)

TF encompass the perceived usefulness, ease of use (Düzgün and Çelik, 2023; Yan and Yu, 2023). In the TAM, both perceived usefulness and perceived ease of use are hypothesized as direct antecedents influencing users’ behavioral intention and actual usage. However, in the context of contemporary AI applications, the distinction between these two constructs may become blurred. However, in current artificial intelligence technologies, ease of use may be highly integrated with usefulness, allowing users to obtain practical value without particularly complex operations. Users can effortlessly derive significant practical utility—such as one-click lesson plan generation and intelligent homework grading—without engaging in complex operational procedures. This integration challenges the traditional conceptual separation. Therefore, this study proposes to consolidate perceived usefulness and perceived ease of use into a unified higher-order construct, termed “Technological features” to more accurately reflect the user experience with modern AI tools. When AI can effectively integrated into teachers’ instructional processes, they can significantly alleviate technostress and enhance teachers’ overall comfort and confidence in technology use (Qi and Zhao, 2024).

Therefore, this study makes the hypothesis:

*H3*:The adaptability of TF can effectively alleviate teachers’ TS.

#### 2.5.4 Organizational support (OS)

OS comprising institutional promotion policies, accessible technological resources, and effective training programs—can significantly influence teachers’ levels of technostress (Trua et al., 2023; Dong et al., 2020; Estrada-Muñoz et al., 2020). Support for technology use not only enhances teachers’ perceptions of technological features but also effectively alleviates technostress among K–12 teachers. Furthermore, research indicates that K–12 teachers’ decisions regarding whether to adopt technology are strongly influenced by the degree of organizational support available to them (Zhang and Guo, 2023).

This study makes the hypothesis

*H4.1*: OS can effectively alleviate teachers’ TS.

*H4.2.1*: OS can effectively enhance teachers’ DA.

*H4.2.2*: OS can effectively enhance DTKS.

*H4.2.3*: OS can effectively enhance DAC.

*H4.3*: OS can effectively alleviate teachers’ RC.

*H4.4*: OS can effectively enhance teachers’ perception of TF.

### 2.6 Hypothetical model

**Figure 1:**
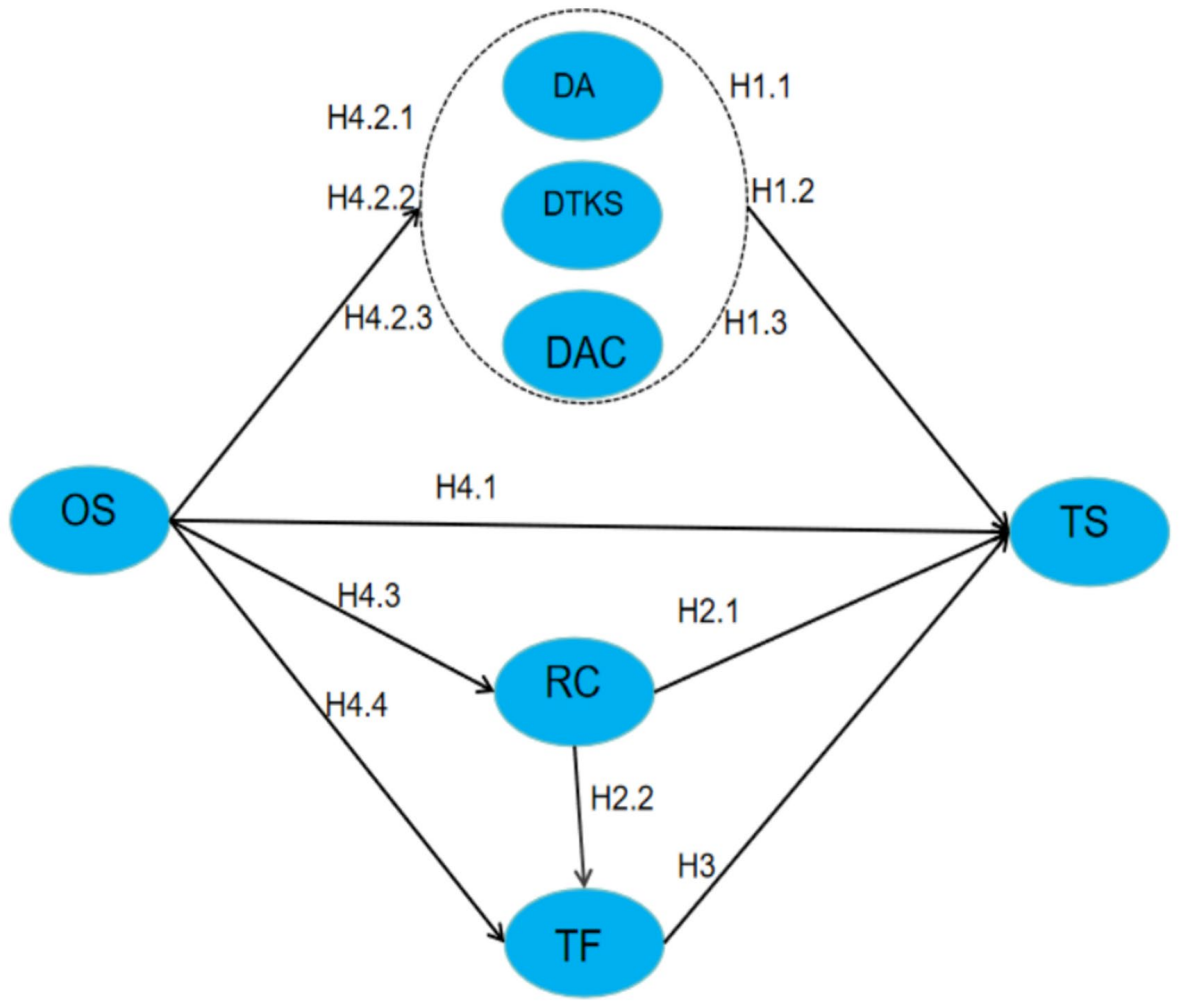
The hypothetical model.

The hypothetical model of this study is shown in Figure 1 below.

## 3 Method

### 3.1 Participants

Due to the absence of a clearly defined sampling frame and constraints associated with research costs, this study adopted a convenience sampling approach. Online questionnaires were distributed to middle schools in Northwest China, primarily focusing on Shaanxi Province, through SOjump,^1^ a widely used online survey platform in China. After completing the questionnaire, participating teachers were encouraged to share the survey link with their colleagues. Prior to participation, all respondents had completed the regional-level *Information Technology Application Ability Improvement Project 2.0* and had experience using relevant artificial intelligence (AI) tools. Data were collected between May 7, 2024, and June 20, 2024. All teachers participating in the current study voluntarily provided informed consent to participate via the online survey. The questionnaire instructions explicitly delineated that upon submitting their informed consent for study participation, the respondents were fully apprised that their personal identifiable information would be subject to strict confidentiality protocols, and the de-identified survey data would be securely stored in a self-constructed database with restricted access.

In total, 318 online questionnaires were collected. After excluding five responses with excessively short completion times and twelve responses containing outliers, 301 valid questionnaires were retained, resulting in a final valid response rate of 94.7%. Most non-respondents were primarily due to random circumstances such as temporary teaching assignments or time conflicts, which were unrelated to the core variable. Thus, no additional measures were taken to address non-response bias. The demographic characteristics of the participants are presented in Table 1.

**Table 1:** Demographic statistics.

Demographics	Variant	Quantity	Percent (%)
Gender	Male	135	44.7
	Female	166	55.3
Educational background	Junior college degree	4	1.33
	Undergraduate degree	183	61
	Master and Doctor Degree	114	37.67
Teaching experience	Within 5 years	40	13.33
	5 ~ 8 years	46	15
	8 ~ 15 years	55	18.33
	More than 15 years	160	53.33
Location of school	Capital city	115	38
	General city	38	12.67
	County area	32	10.67
	Towns and villages	116	38.67
School ownership	Public	286	95.02
	Private	15	4.98

### 3.2 Research instrument

Because some of the original questionnaire items were written in English, and all participants in this study were Chinese, the back-translation technique was employed to ensure the linguistic validity and cultural applicability of the Chinese version. Subsequently, three professors specializing in Educational Technology were invited to review and evaluate the questionnaire. Based on their feedback, the questionnaire’s structure and wording were refined. A pilot test was then conducted with 40 middle school teachers to further assess the instrument’s clarity and reliability. Following the analysis of pilot feedback, one item—“*We are always experiencing new updates and developments in the digital technologies used in our teaching*”—was removed from the final version of the questionnaire.

*The questionnaire is composed of two main sections: basic information and the measurement scale. The basic information section collects demographic data, including gender, educational background, teaching experience, and school location. The measurement scale comprises 40 items in total. All items are rated on a five-point Likert scale, where scores from 1 to 5 correspond to “strongly disagree,” “disagree,” “neutral,” “agree,” and “strongly agree.”*

The PC of teachers in this study were primarily derived from the Digital Literacy of Teachers framework issued by the Ministry of Education of China, which provides a comprehensive foundation for assessing teachers’ digital literacy (Ministry of Education of the People’s Republic of China, 2022). Accordingly, the questionnaire was developed based on the framework’s definitions and dimensions, encompassing *DA*, *DTKS*, and *DAC.*

The DA dimension includes sample items such as: “*I can recognize that applying digital technology resources in teaching requires innovations in teaching theories, models, and methods” and “I proactively learn about the functions and roles of digital technology resources.”* The DTKS dimension includes items such as: *“I understand the concepts and operating principles of common digital technologies such as big data, virtual reality, and artificial intelligence.”* The DAC dimension includes items such as: *“I can design teaching activities that integrate digital technology resources based on instructional objectives.”*

In total, the DA dimension contains 10 items, DTKS contain 6 items, and DAC contains 8 items.

The RC dimension was developed based on two sources: *the occupational nature dimension of the Teacher Technostress Scale proposed by* Coklar et al. (2017), and scholarly interpretations of teachers’ digital literacy in the digital age as discussed by Wu et al. (2023). This dimension includes four items. Example items include: *“I believe that teaching students digital technology and enhancing their digital literacy-related capabilities fall outside the scope of my teaching duties” and “The use of digital technology involves considerable uncertainty, and I am concerned that it may undermine my authority in the classroom.”*

The OS and TS dimensions were adapted from the questionnaire developed by Dong et al. (2020), which demonstrated high reliability, with a Cronbach’s alpha of 0.91. The OS dimension consists of five items. Example items include: *“The school and relevant departments are concerned about the difficulties we encounter during digital teaching and assist in resolving them” and “The school has arranged for professional technical personnel to support our use of digital technologies and effectively help solve problems during their application.”*

The TS dimension consists of four items, including: *“The requirement to use* var*ious digital technologies regularly as part of digital transformation makes me feel uncomfortable”* and “I feel uneasy when using digital technologies during the teaching process.”

The TF dimension was developed primarily based on the constructs of perceived usefulness and perceived ease of use from the TAM. This dimension includes two items, such as: *“The operation of new digital technologies is too complex for me to understand and use quickly and effectively”* and *“The existing digital technology applications cannot be effectively integrated into classroom instruction to enhance my teaching.”*

Reliability testing of the questionnaire was conducted using SPSS 21.0. Following the pilot test, the item related to the frequency of technological updating was removed to improve consistency. The revised questionnaire demonstrated strong reliability, as shown in Table 2, indicating that the instrument possesses a sound structural design.

**Table 2:** The standardized loadings, CR, AVE, and Cronbach’s alpha of the model.

Items	Standardized loadings		CR	AVE	α
DA	DA1	0.7	0.918	0.722	0.922
	DA2	0.936			
	DA3	0.902			
DTKS	DTKS1	0.913	0.953	0.846	0.932
	DTKS2	0.93			
DAC	DAC1	0.916	0.972	0.858	0.972
	DAC2	0.97			
	DAC3	0.91			
OS	OS1	0.877	0.972	0.807	0.956
	OS2	0.927			
	OS3	0.948			
	OS4	0.892			
	OS5	0.837			
RC	RC1	0.776	0.897	0.672	0.789
	RC2	0.812			
	RC3	0.893			
	RC4	0.327			
TF	TF1	0.765	0.831	0.555	0.711
	TF2	0.723			
TS	TS1	0.858	0.947	0.751	0.931
	TS2	0.907			
	TS3	0.868			
	TS4	0.824			

After these modifications, both the individual dimensions and the overall questionnaire demonstrated good internal consistency. The Cronbach’s *α* coefficients for the questionnaire dimensions ranged from 0.711 to 0.972, indicating satisfactory reliability.

Regarding construct validity, the Kaiser–Meyer–Olkin (KMO) test and Bartlett’s test of sphericity were conducted. The results showed that the KMO value of the sample data was 0.743, and the approximate chi-square value for Bartlett’s test was 1106.993 (*p* = 0.000 < 0.001). These results indicate that the dataset meets the statistical requirements for factor analysis and that the questionnaire items are suitable for such analysis.

Convergent validity is demonstrated in two ways: first, the factor loadings must be statistically significant and exceed 0.5; second, the Average Variance Extracted (AVE) for each factor should be greater than 0.5 (Fornell and Larcker, 1981). In this analysis, the item RC4 (“I feel my understanding of education will change due to the use of digital technology”) had a loading below 0.5 and was therefore deleted. Subsequently, it was found that the AVE for the ‘Facilitating Conditions’ construct was below the 0.5 threshold. However, Fornell and Larcker (1981) noted that an AVE below 0.5 can still be considered adequate if the construct’s Composite Reliability (CR) is higher than 0.6. The reliability of the scale is confirmed as the CR indices for all constructs were indeed higher than 0.6 (Bagozzi and Yi, 1988).

### 3.3 Data collection

Data collection was carried out using the online survey platform *SOjump* (see text footnote 1, accessed on June 30, 2024). Ethical approval for this study was obtained from the university’s Ethics Review Board, and all procedures adhered to the ethical principles outlined in the Declaration of Helsinki. Prior to completing the questionnaire, all participants provided informed consent and voluntarily agreed to participate in the survey. The survey was fully anonymous, with no collection of personally identifiable information. Prior to data collection, all participants were afforded informed consent, comprising explicit information regarding the study’s objectives, participant responsibilities, and the voluntary basis of engagement—they reserved the unconditional right to withdraw at any stage without repercussions. Rigorous protocols were enacted to uphold anonymity and confidentiality, with de-identified survey data encrypted and securely archived. The research presented no foreseeable risks of harm to participants and centered solely on the technostress experiences of primary and middle school teachers.

### 3.4 Data analysis

The analytical process was carried out in three stages. First, confirmatory factor analysis (CFA) was conducted to assess the validity of the research instrument. According to Byrne (2010), the total sample size for CFA should be five to ten times the number of items in the scale, and the current sample meets this requirement. CFA was performed using AMOS 21.0. Following the recommendations of Schumacker and Lomax (2016), construct validity was evaluated using the standardized regression weights of the measurement items, composite reliability (CR), average variance extracted (AVE), the square root of the AVE, and model fit indices. In line with Byrne’s (2010) guidelines, the goodness-of-fit indices for CFA included the Tucker–Lewis Index (TLI), standardized root mean square residual (SRMR), normed fit index (NFI), comparative fit index (CFI), and root mean square error of approximation (RMSEA).

Second, structural equation modeling (SEM) was employed to construct the influencing factor model, with parameter estimation conducted using AMOS 21.0. Initial model fit was assessed using five key indices: Chi-Squared to Degrees of Freedom Ratio (CMIN/DF), CFI, TLI, RMSEA, and SRMR. Subsequent to the model modification guided by modification indices (MI), the fit of the revised structural model was re-evaluated using the same set of fit indices.

In line with Hu and Bentler (1999), the following cutoff criteria were adopted to indicate a good model fit: SRMR ≤ 0.080, RMSEA ≤ 0.060, TLI ≥ 0.900, and CFI ≥ 0.900.

To examine the relationships among the variables in this study, Pearson correlation analysis was conducted, with the results presented in Table 3 below. No significant correlations were identified between TF and DA, TF and DTKS, TF and DAC, TS and DTKS, TS and DAC, or TS and OS. In contrast, significant correlations were observed among all remaining variables.

**Table 3:** Correlations among variables.

Study variable	1	2	3	4	5	6	7
1. DA	1						
2. DTKS	0.696**	1					
3. DAC	0.611**	0.851**	1				
4. OS	0.487**	0.560**	0.590**	1			
5. RC	0.261**	0.475**	0.552**	0.534**	1		
6. TF	−0.051	−0.056	−0.021	−0.249**	−0.390**	1	
7. TS	−0.138*	−0.078	−0.089	0.080	0.390**	0.663**	1
Mean (x)	4.2807	3.7645	3.6884	3.4348	2.9672	2.9061	2.5930
Standard deviations	0.66661	0.91154	0.88778	0.93106	0.87665	0.97515	1.00197

***P < 0.001; **p < 0.01; *p < 0.05.

We evaluated model fit via RMSEA (with 90% confidence interval, CI): The Default model had an RMSEA of 0.056 (90% CI [0.048, 0.064]), satisfying the criterion for good fit (RMSEA < 0.060).

The Independence model showed a high RMSEA of 0.291 (90% CI [0.284, 0.297]), which is expected (it serves as a poor-fit benchmark).

The model fitting index are presented in Table 4, all fit indices met the required criteria and fell within acceptable ranges.

**Table 4:** Model fitting index.

Fitting index	SRMR	CFI	RMSEA	TLI	CMIN/DF
Standard value	<0.080	>0.90	<0.060	≥0.900	<5.00
Measured value	0.071	0.967	0.056	0.963	1.977

## 4 Results

### 4.1 The technostress level of middle school teachers

The level of technostress among middle school teachers is presented in Table 4. The mean value is 2.593, with a standard deviation of 1.00197.

Regarding gender, the study found that male teachers experience higher levels of technostress than female teachers. Analysis across various dimensions revealed no significant gender differences in personal characteristics, organizational support, or technological characteristics, which aligns with findings from previous studies. However, a significant difference was observed in the overall level of technostress.

With respect to teaching experience, teachers with 5–8 years of experience and those with more than 15 years of experience reported significantly higher levels of technostress.

In terms of school location, technostress levels increased progressively from teachers in provincial capitals to those in rural areas.

Concerning educational background, the results indicated that teachers’ technostress decreases gradually with higher academic qualifications. The primary source of stress was identified as role conflict, with significant disparities observed. These differences are mainly reflected in teachers’ varying perceptions of their roles and responsibilities within the context of human–machine collaboration.

In terms of school ownership, the sample of this study is predominantly concentrated in public middle schools and lacks diversity in this dimension. Thus, no in-depth discussion will be provided for this aspect.

### 4.2 Hypothesis testing

According to the reference standards for interpreting AMOS research results, a |*β*| value greater than 0.1 indicates a meaningful effect (Wu, 2010). The model of technostress influencing factors was tested and analyzed, and the standardized path coefficients and corresponding *p*-values for each hypothesized relationship were obtained, as presented in Table 5.

**Figure 2:**
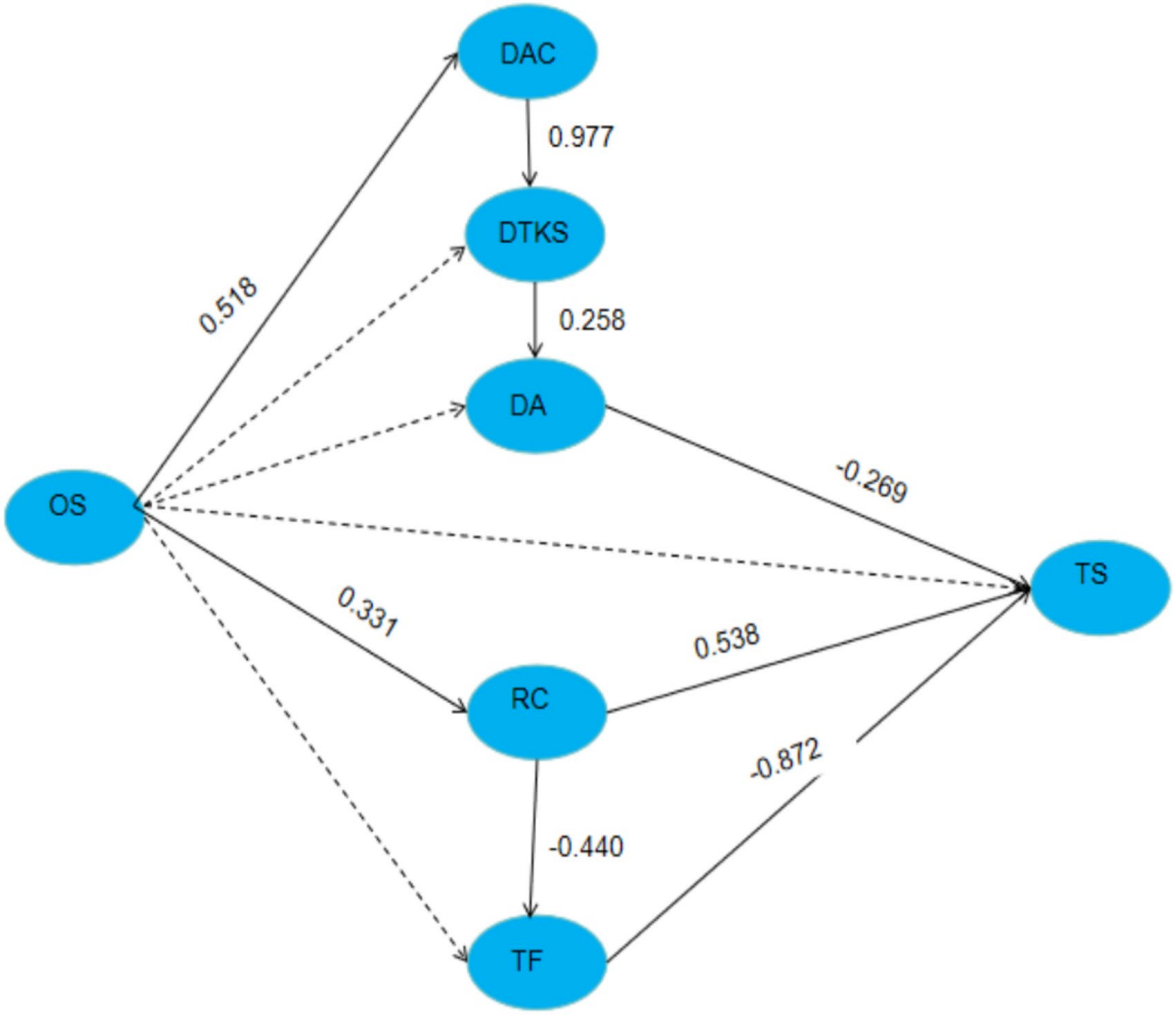
The final study.

(1) Paths ‘DA-TS’, ‘RC-TS’, ‘TF-TS’, ‘RC-TF’, ‘OS-IC’, ‘OS-RC’, all reach a significant level, indicating hypothesis H1.1, H2, H3, H4.2.3, H4.3 are valid.
(2) The *p* value of path ‘DTKS-TS’, ‘DAC-TS’, ‘OS-TS’, ‘OS-TF’ is greater than 0.05, the |β| of ‘OS—DA’, ‘OS — DTKS’ indicating hypothesis H1.2, H1.3, H4.1, H4.2.1, H4.2.2, H4.4 are not valid, further analysis these factors reveals that digital application competence and digital technology knowledge and skills are significantly correlated (|β| = 0.988, *p* < 0.001), digital technology knowledge and skills and digital awareness are significantly correlated (|β| = 0.282, *p* < 0.001).
(3) The direct, indirect and total effects of technology features, digital awareness, role conflict and organizational support on technostress among teachers are shown in Table 6 below.
(4) The study evaluated and tested the structural relationship of the constructs, as shown in Figure 2.

**Table 5:** Hypothesis test result.

Hypothesis	Hypothetical path	β	SE	CR	Results
H1.1	DA—TS	−0.257**	0.92	−2.785	Support
H1.2	DTKS—TS	0.125	0.111	1.124	Disupport
H1.3	DAC—TS	−0.202	0.121	−1.665	Disupport
H2.1	RC—TS	0.152*	0.063	2.392	Support
H2.2	RC—TF	−0.437	0.062	−7.091	Support
H3	TF—TS	−0.872***	0.107	−8.133	Support
H4.1	OS—TS	−0.117*	0.050	−2.322	Disupport
H4.2.1	OS—DA	0.063*	0.030	2.110	Disupport
H4.2.2	OS—DTKS	0.020	0.039	0.512	Disupport
H4.2.3	OS—DAC	0.524***	0.045	11.741	Support
H4.3	OS—RC	0.350	0.082	4.263	Support
H4.4	OS—TF	−0.074	0.054	−1.364	Disupport

	β	S.E.	C.R.	*P*
DTKS ← DAC	0.988	0.056	17.731	***
DA ← DTKS	0.282	0.063	4.450	***

***P < 0.001; **P < 0.01; *P < 0.05.

**Table 6:** Direct and indirect effect analysis.

Dependent variable	Independent variable	Direct effects	Indirect effects	Total effects
TS	TF	−0.872	0.000	−0.872
	DA	−0.269	−0.000	−0.269
	RC	0.154	0.384	0.538
TF	RC	−0.440	0.000	−0.440
RC	OS	0.320	−0.010	0.331
DA	DTKS	0.258	0.000	0.258
DTKS	DAC	0.977	0.000	0.977
DAC	OS	0.518	0.000	0.518

TF exerted a significant direct negative effect (−0.872) on TS with no indirect effects observed.

DA exerted a significant direct negative effect (−0.269) on TS with no indirect effects observed.

In contrast, RC exhibited a direct positive effect (0.154) and an indirect positive effect (0.384) on TS, resulting in a total effect of 0.538.

RC exerted a significant direct negative effect (−0.440) on TF with no indirect effects observed.

OS primarily influences teachers’ digital literacy through direct mechanisms. Specifically, OS exerts a direct positive effect (0.518) on DAC, which in turn has a strong positive effect (0.977) on digital technology knowledge and skills (DTKS). DTKS further demonstrates a direct positive effect (0.258) on DA.

The relationship between OS and RC was confirmed; however, the study unexpectedly found that OS exacerbated TS among middle school teachers (0.331).

Collectively, these results reveal a multidimensional influence mechanism between organizational support (OS) and technological stress (TS), which operates through three pathways: “OS → DAC → DTKS → DA → TS,” “OS → RC → TS” and “OS → RC → TF → TS.”

## 5 Discussion

### 5.1 Demographic characteristics and technostress

The study found middle school teachers’ levels of technostress were relatively low but varied significantly across demographic groups. Male teachers exhibited higher levels of technostress than female teachers, consistent with the findings of Estrada-Muñoz et al. (2020). However, the study did not find statistically significant differences.

Regarding teaching experience, teachers with 5–8 years of experience and those with more than 15 years of experience reported higher levels of technostress. For teachers with 5–8 years of experience, stress primarily stemmed from the need to adapt their established teaching practices to the integration of AI technologies. In contrast, for teachers with over 15 years of experience, technostress was mainly associated with the application and continuous learning of digital resource platforms. These findings align with the stages of technology acceptance described in the SAMR model, suggesting that different phases of technology adoption correspond to distinct sources of technostress (Song et al., 2025).

In terms of school location, teachers’ technostress increases progressively from provincial capitals to rural areas. Analysis across different dimensions indicates that the most significant differences lie in organizational support, which warrants greater attention during the implementation of educational technologies in rural primary and middle schools. This finding suggests that rural teachers continue to face practical challenges in applying various digital technologies, highlighting the need to strengthen institutional and technical support for rural schools (Zhang and Guo, 2023).

Regarding educational background, the study found that teachers’ technostress decreases gradually with higher academic qualifications. The primary sources of stress are concentrated in role conflict, with significant disparities observed among teachers of different educational backgrounds. These disparities mainly stem from differences in how teachers perceive their roles and responsibilities in the context of human–machine collaboration. This suggests that enhancing teachers’ academic qualifications can deepen their understanding of pedagogy and technology integration, thereby reducing technostress.

### 5.2 Personal competence of teachers

DA was found to significantly reduce teachers’ technostress, hypothesis H1.1 was supported, a result consistent with the findings of Panisoara et al. (2020), Dong et al. (2020), and Zhao (2012). In particular, in the three dimensions of DA, digital willingness plays a crucial role in alleviating technostress. Teachers with strong motivation and willingness to engage with digital technologies tend to hold firmer beliefs in the potential of technology to enhance classroom teaching and promote professional growth (Zhou et al., 2024).

In contrast, DTKS and DAC did not significantly alleviate teachers’ technostress, H1.2 and H1.3 were not supported, which differs from the conclusions of previous studies. This is because, during the early stages of digital adoption, teachers often engage only in simple, low-risk tasks that require minimal adjustment to existing teaching practice, the current requirements for digital knowledge, skills, and application abilities are relatively low. This further indicates that the influence of teachers’ motivations on technostress is more significant than that of their technical knowledge level or skill mastery (Fang and Zhang, 2024).

Nevertheless, DTKS can indirectly influence technostress by enhancing teachers’ DA, while DAC can substantially strengthen DTKS. This pattern reflects a positive transfer mechanism of “practice → knowledge → awareness” in mitigating technostress.

### 5.3 Role conflict

Role conflict was found to significantly increase teachers’ technostress, hypothesis H2.1 was supported, consistent with the findings of Fang and Zhang (2024). An in-depth analysis revealed that the primary source of teachers’ current role conflict arises from an increased workload. With the integration of AI, the relationship between teacher and students is facing reshaping. Teachers not only need reconstruct their own knowledge systems, but also strive to coordinate the tripartite relationship among “teachers, students, and technology” (Li and Luo, 2019). In parallel, societal expectations pertaining to the competent, context-appropriate application of AI technologies by teachers in instructional practice have grown steadily. Teachers’ professional workload has thus been escalated, with the technostress of middle school teachers being subjected to significant intensification, furthermore, role conflict can also weaken secondary school teachers’ perception of technological characteristics and indirectly increase their technostress, hypothesis H2.2 was supported.

### 5.4 Technological features

Appropriate TF can significantly alleviate teachers’ TS, hypothesis H3 was supported, consistent with findings from previous studies (Christian et al., 2020; Califf and Brooks, 2020). On the one hand, AI technologies applied in educational contexts remain in a developmental stage, often resulting in a gap between AI-driven teaching practices and theoretical expectations. On the other hand, most frontline teachers are still in the exploratory phase of integrating AI into practical teaching in innovative ways. Consequently, the provision of stable and user-friendly technological applications can effectively reduce teachers’ technostress (Zhang and Gu, 2023; Can and Nguyen, 2025).

### 5.5 Organizational support

Organizational support demonstrated a dual-mechanism effect on technostress. On the one hand, it alleviate technostress through a positive transmission chain—“DAC → DTKS → DA”; on the other hand, it indirectly amplifies technostress through the mediating effect of RC, meanwhile, it weakens middle school teachers’ perception of TF through the exacerbation of role conflict.

*Organizational support cannot effectively enhance teachers’ perception of technological features, hypothesis H4.4 was disupported, the finding diverge markedly from the anticipated outcomes posited in the research hypotheses. This is because* AI applications remain in their exploratory stage, with the technology itself continuing to evolve. In classroom practice, teachers typically employ only basic AI functionalities, and the deeper pedagogical potential of AI has yet to be fully realized. Consequently, research and implementation in this field remain relatively superficial and fragmented, limiting the full display of AI’s advantages and hindering its innovative diffusion in education (Rao et al., 2025).

OS can effectively enhance teachers’ DAC, thus hypothesis H4.2.3 was supported. In contrast, it exerts no direct impact on DTKS or DA, meaning hypotheses H4.2.1 and H4.2.2 were not supported. This is because current organizational support measures—such as AI-related training—are primarily focused on the pedagogical application of AI technologies rather than the core technical aspects themselves. The study also found that the improvement of teachers’ DAC can effectively promote their DTKS, which in turn enhances their DA.

OS cannot directly alleviate teachers’ technostress, hypothesis H4.1 was disupported, the finding diverges from previous research. This is because the hierarchical nature of digital transformation policies, in which mandatory requirements for digital development are passed down through multiple administrative levels to individual teachers. As a result, teachers often find themselves in a passive position, compelled to acquire additional knowledge and skills, thereby increasing their workload and psychological burden, furthermore, since current AI applications are still in the initial stage of exploration, teachers’ level of dependence on organizational application support is not particularly high (Christian et al., 2020; Song and Wu, 2023).

*The relationship between organizational support and role conflict has been verified. However, surprisingly, the study found that organizational support not only failed to alleviate middle school teachers’ role conflict but also exacerbated it, leading to the partial support of Hypothesis H4.3*. On the one hand, the generalization or detachment of role expectations inherent in organizational support—coupled with the lack of clear role norms, detailed descriptions, and accurate interpretations—has resulted in deviations in teachers’ understanding and cognition of these role expectations. On the other hand, middle school teachers’ own role transformation remains constrained and hindered by traditional role fixation and role inertia; they are reluctant to abandon conventional roles and construct new ones that align with the requirements of the digital transformation of education (Luo and Wu, 2025).

## 6 Conclusion

Grounded in the TAM, this study integrates factors such as organizational support, teachers’ personal attributes, and role conflict to examine the determinants of technostress among middle school teachers within the context of AI-enabled education, as well as the mechanisms underlying their interactions. This research contributes to the theoretical understanding of technostress in educational settings by extending the application of TAM to the digital-intelligence era. The findings reveal that, across different groups of teachers, similar levels of technostress may originate from distinct sources.

Using structural equation modeling (SEM), this study verified a multidimensional mechanism underlying teachers’ technostress within AI-enabled educational contexts. Specifically, technological features emerged as the core proximal predictor, exerting a significant direct negative effect (−0.872) on technostress.

Role conflict was identified as the primary risk factor, with a total effect of 0.538. It not only exerted a direct positive effect on technostress (0.154), but also indirectly increased stress by diminishing teachers’ perceptions of technological features (0.440).

In contrast, digital awareness functioned as a key protective factor, reducing technostress through a direct pathway (−0.269). Although organizational support did not have a significant direct effect on technostress, it alleviated stress indirectly through the sequential pathway “digital application competence → digital knowledge and skills → digital awareness.”

Notably, organizational support exhibited a positive overall influence on role conflict (0.331). This finding suggests that the design and implementation of organizational support require further optimization to better align with teachers’ evolving roles and responsibilities in AI-integrated education.

### 6.1 Adopt a tiered, category-based strategy to provide precise support

To mitigate technostress among middle school teachers in AI-enabled educational environments, it is crucial to establish a tiered, category-based, and role-specific training system. The training content should be thoughtfully designed and aligned with teachers’ professional stages and the practical challenges they encounter (Tian et al., 2024). For instance, targeted training and support related to technical operations should be prioritized for teachers with longer teaching experience, while for younger teachers, the focus should be on the effective integration of AI technologies with teaching and learning. For relatively remote rural areas, greater support should be provided in terms of policy promotion and technical accessibility, while urban teachers should focus more on their own role adaptation. Additionally, organizational support needs to be dynamically adjusted based on teachers’ level of technology acceptance to effectively mitigate technostress (Lin et al., 2025).

### 6.2 Starting from actual needs, balance the supply–demand relationship

In the early stage of AI integration into education and teaching, technological features represent a key factor influencing teachers’ technostress. At present, most middle school teachers remain in the initial phase of adopting emerging technologies such as AI, and the educational advantages of AI have yet to be fully realized. Previous studies have also indicated that many existing AI technologies face challenges in being effectively integrated into classroom practice (Li and Shi, 2020).

Therefore, future efforts to integrate AI into education should begin with the intrinsic needs of teachers and students, focusing on optimizing the functionality and usability of relevant platforms. Simultaneously, developing tiered and age-appropriate standards for AI-supported teaching should be prioritized. In alignment with students’ cognitive development and age characteristics, it is essential to establish clear application strategies and implementation guidelines for AI across diverse instructional scenarios, supported by age-adapted user manuals and practical case examples (Kong et al., 2025).

### 6.3 Building on the use of typical cases helps enhance teachers’ digital awareness

Teachers’ technostress in the context of digital transformation is situational in nature (Zhao, 2020). During the early phase of AI implementation in education, teachers’ perception of technology as a tool that enhances their work is the primary motivator for adoption—particularly among those still resistant to AI integration. Therefore, effectively alleviating technostress and promoting AI adoption among middle school teachers requires the development of practical use cases for generative AI applications and the provision of continuous, practice-oriented training. For example, the University of Pennsylvania suggests that instructors utilize AI tools to provide multiple examples and explanations for complex or abstract concepts to enhance teaching effectiveness; Cornell University supports teachers in leveraging AI to generate initial drafts of syllabi, graphics and charts, demonstration experiments, and simulated scenarios. As a result, this can save instructors’ time and effort, thereby improving teaching efficiency (Jiang and Hu, 2025). By engaging with authentic use cases, teachers can better understand and personally experience the transformative potential of AI in education. This process not only strengthens their awareness of technology’s value but also enhances their motivation and confidence to integrate AI into teaching practice—thereby reducing technostress and fostering sustainable technology acceptance.

### 6.4 Strengthen role specification and flexibility to ease teachers’ role conflict

AI’s human-like cognitive capabilities have introduced a new paradigm of human–AI collaboration, which presents significant challenges for middle school teachers who are accustomed to traditional pedagogical practices (Yuan and Liu, 2024). Therefore, to address these challenges, when promoting artificial intelligence technology, organizations must first set realistic role expectations for teachers based on their actual circumstances. Secondly, it is essential to clarify role norms for teachers and foster a positive public opinion environment. Simultaneously, a multi-dimensional support system should be built through measures such as flexible work arrangements, clear role definitions, and positive public guidance. This will facilitate the orderly transformation of teachers’ roles and ensure the deep integration and effective implementation of AI technology in the field of education.

## 7 Limitations

This study explored the factors influencing middle school teachers’ technostress in the context of AI integration, as well as the mechanisms through which these factors interact, with the goal of proposing effective strategies to alleviate teachers’ technostress. However, several limitations should be acknowledged.

First, due to constraints in research capacity, data were primarily collected from middle schools in underdeveloped regions of China—specifically, the northwestern area (mainly Shaanxi Province). The research sample exhibits distinct regional characteristics, as these regions are still in the early stages of promoting artificial intelligence technology, the findings may not be fully generalizable to all secondary school teachers across different parts of the country; Second, this study focuses on the technostress among middle school teachers and did not conduct specific statistical analyses regarding the socioeconomic backgrounds or urban–rural distribution of the research subjects; although the institutional affiliation (public vs. private schools) was recorded, no in-depth discussion was undertaken based on these dimensions due to the uneven sample distribution; Third, this study did not conduct a longitudinal analysis of technostress development or examine how influencing factors change over time.

In light of these limitations, future research should expand the sample size and geographical scope to improve the generalizability of findings; meanwhile, longitudinal and categorized investigations should be conducted to trace how teachers’ technostress evolves alongside changes in technology adoption and pedagogical adaptation. Such research will enable the formulation of more targeted and evidence-based strategies for mitigating teachers’ technostress in the era of AI-enabled education.

## Data Availability

The datasets presented in this study can be found in online repositories. The names of the repository/repositories and accession number(s) can be found at: https://www.wjx.cn/report/266239079.aspx?sat=1.

## Footnotes

1
https://www.wjx.cn/
